# A novel circular RNA circENTPD7 contributes to glioblastoma progression by targeting ROS1

**DOI:** 10.1186/s12935-020-01208-9

**Published:** 2020-04-10

**Authors:** Fei Zhu, Cheng Cheng, Hong qin, Hongsheng Wang, Hailong Yu

**Affiliations:** Department of Neuro Surgery, The Affiliated Hospital of Yangzhou University, No. 45, Taizhou Road, Yangzhou, Jiangsu China

**Keywords:** circRNA, Glioblastoma, circENTPD7, ROS1, miR-101-3p

## Abstract

**Background:**

Circular RNAs (circRNAs) are identified to play an important role in many human cancers, such as glioblastoma. However, the potential mechanisms underlying the relationship between circRNAs and glioblastoma pathogenesis are still elusive. This study is designed to investigate the role of circRNAs in glioblastoma progression.

**Methods:**

The present study is designed to investigate the mechanism by which circRNAs involves in glioblastoma pathogenesis. By using circRNAs microarray, we detected the dysregulated circRNAs and identified an up-regulated circRNA, circENTPD7 in glioblastoma tissues. Cell proliferation was measured using a CCK-8 assay. Cell clone formation ability was assessed with a clone formation test. We used the bioinformatics website to predict circRNA–miRNA and miRNA–mRNA interactions. CircRNA–miRNA interaction was confirmed by dual-luciferase reporter assays and RNA–RNA pulldown assay.

**Results:**

circENTPD7 (hsa_circ_0019421) was upregulated in glioblastoma tissues. Kaplan–Meier survival analysis indicated that glioblastoma patients had a poor overall survival when circENTPD7 expression levels were high. Knockdown of circENTPD7 inhibited the motility and proliferation of glioblastoma cells. Moreover, we demonstrated that circENTPD7 acted as a sponge of miR-101-3p to regulate the expression of ROS1 further promoted the proliferation and motility of glioblastoma cells.

**Conclusions:**

Taken together, these findings indicate that circRNA circENTPD7 promotes glioblastoma cell proliferation and motility by regulating miR-101-3p/ROS1.

## Background

Glioblastoma is the most common neurologic cancer in worldwide and is the top leading cause of neurologic cancer-related death worldwide [1]. Tumor metastasis is a common and major obstacle to improve the survival of patients with glioblastoma [2]. Despite recent advances in surgery, chemotherapy and molecular targeted therapies, glioblastoma still has poor morbidity and mortality [3, 4]. The challenge of treating glioblastoma includes not only tumor metastasis and recurrence, but also uncertain and non-specific therapeutic targets [5, 6]. A better understanding of glioblastoma pathogenesis is critical for advancing and improving available therapeutic markers and targets [7].

Circular RNAs (circRNAs), a new subtype of non-coding RNAs, are covalently closed loop RNAs formed by 3′ end to 5′ end joining RNA fragments [8]. Although circRNAs had been identified for more than four decades, they had only received attention in recent years [9]. Using high-throughput sequencing approach, more than 30,000 circRNAs had been identified. CircRNAs are ubiquitous expressed in many tissues including glioblastoma tissues [10–12]. Some circRNAs had been investigated in glioblastoma tissues, for instance, hypoxia-associated circDENND2A promoted glioblastoma aggressiveness by sponging miR-625-5p [13]; circular RNA circSCAF11 accelerated the glioblastoma tumorigenesis through the miR-421/SP1/VEGFA axis [14]; EIF4A3-induced circular RNA MMP9 (circMMP9) acted as a sponge of miR-124 and promoted glioblastoma multiforme cell tumorigenesis [11]. These results suggested that cirRNAs play an important role in the development and progression of glioblastoma. However, more circRNAs need to be explored in glioblastoma.

In the present study, we identified a novel circRNA (hsa_circ_0019421), named circENTPD7, which is generated from the ENTPD7 gene locus with spliced length 357 nt. We found that circENTPD7 was upregulated in glioblastoma tissue and cells. Knockdown of circENTPD7 could decrease glioblastoma cell growth and motility. Mechanically, circENTPD7 served as miRNA sponge to decrease miR-101-3p. ROS1 was identified as the target of miR-101-3p. The expression of miR-101-3p and ROS1 in glioblastoma tissues were examined by RT-qPCR. The RNA levels of miR-101-3p were negatively correlated with circENTPD7. Furthermore, miR-101-3p and ROS1 were also involved in glioblastoma cells growth and motility. In collection, these findings indicated that circENTPD7/miR-101-3p/ROS1 signaling pathway provided a new perspective for the treatment of glioblastoma.

## Methods

### Clinical samples

The paired glioblastoma and adjacent normal tissues were collected from the patients at The Affiliated Hospital of Yangzhou University from 2015 to 2019. The specimens were taken after tumor excision within less than 10 min, then the specimens were stored at − 80 °C immediately until application in the experiments. This study was approved by The Ethics Committee of The Affiliated Hospital of Yangzhou University, written informed consents were obtained from all glioblastoma patients.

### Cell culture

All cells were obtained from American type culture collection (ATCC) or The Cell Bank of Type Culture Collection of Chinese Academy of Sciences. All cell lines were authenticated in December 2017 by using short tandem repeat (STR) DNA profiling method. Human glioblastoma cell lines (U87, A172) were incubated in DMEM (Gibco, Grand Island, NY, USA) supplemented with 10% fetal bovine serum (FBS) (Gibco, USA), and 1% penicillin/streptomycin (pen/strep) (Invitrogen, Carlsbad, CA, USA). Cells were incubated in an atmosphere with 5% CO_2_ at 37 °C.

### RNA extraction and quantitative real-time PCR (RT-qPCR)

According to the manufacturers’ instructions, total RNA was obtained from tissues or cells by Trizol reagent (Invitrogen, USA). NanoDrop ND2000 (Thermo Scientific Inc., USA) was used to determine the purity and quantify the concentration of RNA. Total RNA was reverse transcribed by HiScript II Q RT SuperMix for qPCR Kit (Vazyme Biotech Co., Ltd, Nanjing, China). Primers used for RT-qPCR were synthesized by Tsingke Biological Technology (Nanjing, China). According to the manufacturer’s instructions, RT-qPCR was performed using the ChamQ SYBR qPCR Master Mix (Without ROX) (Vazyme Biotech Co., Ltd, Nanjing, China) in a Roche LC 96 qPCR system (Roche, Germany). The PCR reaction started at 95 °C for 2 min, followed by 40 cycles of 95 °C for 10 s, 60 °C for 30 s. Actin or U6 was used as the internal reference of measuring qPCR results. Target gene relative expression levels were measured by 2^−ΔΔCT^ method. The RT-qPCR primers of circENTPD7 are Forward: 5′-ATGCCAGTGATTACCTTCGTC-3′; Reverse: 5′-CTTCAAGCTCCCCTACTCG-3′.

### RNA isolation of nuclear and cytoplasmic fractions

According to manufacturer’s instructions, we employed the NE-PER™ Nuclear and Cytoplasmic Extraction Reagents Kit (Thermo Scientific, USA) to isolate and collect cytosolic and nuclear fractions. The expression levels of GAPDH (cytoplasmic control transcript) and U6 (nuclear control transcript) were examined in nuclear and cytoplasmic fractions using RT-qPCR.

### Cell transfection and viral infection

Lipofectamine 2000 (Life Technologies, USA) was used for plasmid or siRNA transfection. Lentiviral expression systems (psPAX2, pMD2.G and sh- circENTPD7) were generated to transduce glioblastoma cells. For transient knockdown circENTPD7, the small interfering RNAs (siRNAs) were designed and sythesized by GenePharma Co., Ltd. (Shanghai, China). The siRNA sequence crossing the circENTPD7 junction site is: 5′-UCCCUGAGAGGUAUUUGGCU-3′;

### Cell counting kit-8 (CCK-8)

1 × 10^3^ glioblastoma cells were seeded into a 96-well plate. Absorbance at 450 nm was measured after incubating the cells with 100 µL CCK-8 kit (Dojindo Laboratories, Japan) for 1 h.

### Transwell migration assay

8 μm pore size (Millipore, USA) insert was used in this assay. Add 0.5 mL of DMEM with 10% FBS to the lower compartment. Gently add 1 × 10^4^ cells to the insert. The cells were incubated in the transwell plate at 37 °C, 5% CO_2_ for 6 h. Next, we stained the cells with 1% crystal violet. The cells on the lower side of the insert were counted under a microscope.

### Tumorigenesis assay

The mice care and whole experimental protocols were approved by The Affiliated Hospital of Yangzhou University Experimental Animal Welfare Ethics Committee. Animal Experiments were performed in compliance with the guidelines of the Animal Research Ethics Board of Nanjing Medical University (Nanjing, China). 4 weeks old male BALB/c nude mice were purchased from the Charles River laboratories and maintained under pathogen-free conditions during 2018–2019. In the back flank, mice (five in each group) were subcutaneously injected 1 × 10^7^ cells in 200 µL cell suspension. The tumors were measured every week after the tumor was visible and the tumors’ volume were calculated following the formula volume (0.5 × length × width^2^).

### Western blot assay

Proteins were extracted from cells or immunoprecipitation samples using detergent-containing RIPA lysis buffer. Equal amounts of total proteins were subjected to sulphate–polyacrylamide gel electrophoresis (SDS-PAGE) and proteins were transferred to 0.45 μm polyvinylidene difluoride (PVDF) membrane (Millipore, MA, USA). After blocking with 5% non-fat milk, the PVDF membrane was incubated with primary antibodies as follow: anti-ROS1 (abcam, USA), anti-GAPDH (Santa Cruz, USA). Proteins were visualized through horseradish-peroxidase (HRP) conjugated secondary antibody and peroxide LumiGLO reagent system (Cell Signaling Technology, USA).

### Dual-luciferase reporter assay

CircENTPD7 segment (100 bp) or ROS1 3′UTR was constructed into pGL3-control plasmid. Either target sequence or wild-type seed region was co-transfected with 50 ng Ranilla luciferase reporter plasmid into HEK-293T cells that cultured in 48-well plates by using Lipofectamine 2000. The luciferase activities were measured using The Dual-Luciferase^®^ Reporter Assay System (Promega, USA) after 48 h transfection.

### Fluorescence in situ hybridization (FISH)

Cy3-labeled spliced circENTPD7 probe (5′-CTTCTCCCTGAGAGGTATTTGGCTCG-3′) were purchased from RiboBio (Guangzhou, China). The stained cells were photographed via Zeiss Axiovert 200 M laser scanning confocal microscope (Carl Zeiss, Freistaat Thuringen, Germany).

### RNA–RNA pulldown assay

RNA–RNA pull-down assay was employed to detect potential binding between circENTPD7 and miR-101-3p. The biotin-labeled RNA probe targeting circENTPD7 was generated from GenScript Biotech Co., Ltd. (Nanjing, China). The probe sequence was 5′-GCUCCCCUACUCGAGCCAAAUACCUCUCAGGGAGAAGCCUCAUGCCUGCU-3′. The probe was mixed with the lysate of glioblastoma cells for 2 h at 4 °C. Thereafter, the complexes were incubated with streptavidin magnetic beads (Thermo Fisher Scientific, Waltham, MA, USA) for 2 h. At last, the RNA was eluted and the level of circENTPD7-bound miR-101-3p was examined by RT-qPCR.

### Statistical analysis

Values are presented as the mean ± SD. Statistical analysis was evaluated by the unpaired student’s *t* test. Values of *P* < 0.05 were considered to be statistically significant.

## Results

### circENTPD7 is overexpressed in the glioblastoma tissue and cells

**Fig. 1:**
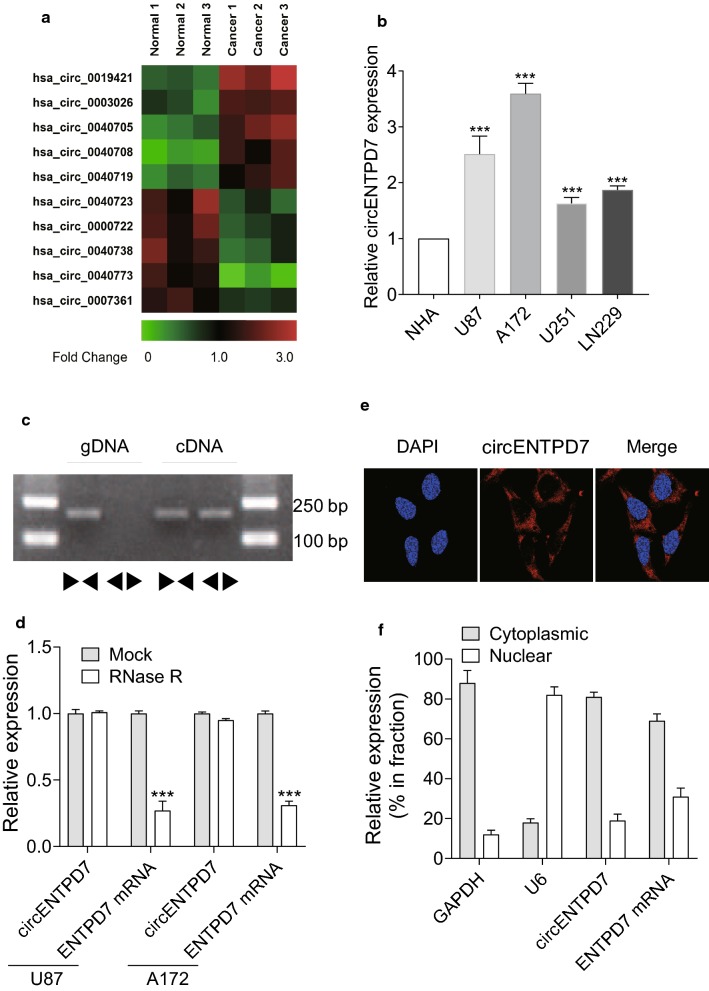
circENTPD7 is overexpressed in the glioblastoma tissue and cells. **a** The heatmap showed the representative dysregulated circRNAs in glioblastoma tissues analyzed by microarray. **b** CircENTPD7 (hsa_circ_0019421) was upregulated in the glioblastoma cells compared to the normal cells. **c** We could only amplify back-spliced forms of ENTPD7 from cDNA by PCR using divergent primers following by gel electrophoresis. The canonical linear forms of ENTPD7 in both and gDNA and were amplified in both cDNA and gDNA. **d** RT-qPCR for the abundance of circENTPD7 and ENTPD7 mRNA in glioblastoma cells treated with RNase R were performed, ENTPD7 mRNA rather than circENTPD7 were decreased after RNase R treatment. **e** CircENTPD7 was most localized in the cytoplasm by FISH analysis in U87 cells. **f** CircENTPD7 was enriched in U87 cytoplasm fraction. Levels of circENTPD7, ENTPD7 mRNA, GAPDH, and U6 RNA in purified U87 nuclear and cytoplasm fractions were detected by RT-qPCR. Data are presented as mean ± SD, Student’s *t* test, ***P < 0.001

To investigate dysregulated circRNAs in glioblastoma, three paired glioblastoma tissues and adjacent normal tissues were analyzed using ArrayStar circRNA microarray. Within the top 100 differently expressed circRNAs, we exhibited five circRNAs that were up-regulated or down-regulated in Fig. 1a. Some of these circRNAs had previously been studied in other types of cancer, however, there are no reports of circENTPD7 (hsa_circ_0019421) in the literature. By using RT-PCR, we examined the expression of circENTPD7 in glioblastoma or normal cells. To compare the circENTPD7 levels between the established cell lines of glioblastoma and normal cells, the circENTPD7 expression was examined in U87, A172 cells. As shown in Fig. 1b, the levels of circENTPD7 in glioblastoma cells were significantly higher than that in normal cells. Using cDNA and genomic DNA (gDNA) from U87 cell lines as templates, the circENTPD7 amplification products were only observed in cDNA by divergent primers but not in gDNA (Fig. 1c). With divergent and convergent primers, we performed RT-qPCR assay and found that circENTPD7, rather than linear ENTPD7 or GAPDH, could resist digestion by RNAse R (Fig. 1d). Additionally, the fluorescence in situ hybridization (FISH) results in U87 exhibited a dominantly cytoplasmic distribution of circENTPD7 (Fig. 1e). Nuclear and cytoplasm fractions were isolated from U87 cells, as shown in Fig. 1f, the isolated cytoplasmic fractions showed a higher level of circENTPD7 than the nuclear fractions.

**Fig. 2:**
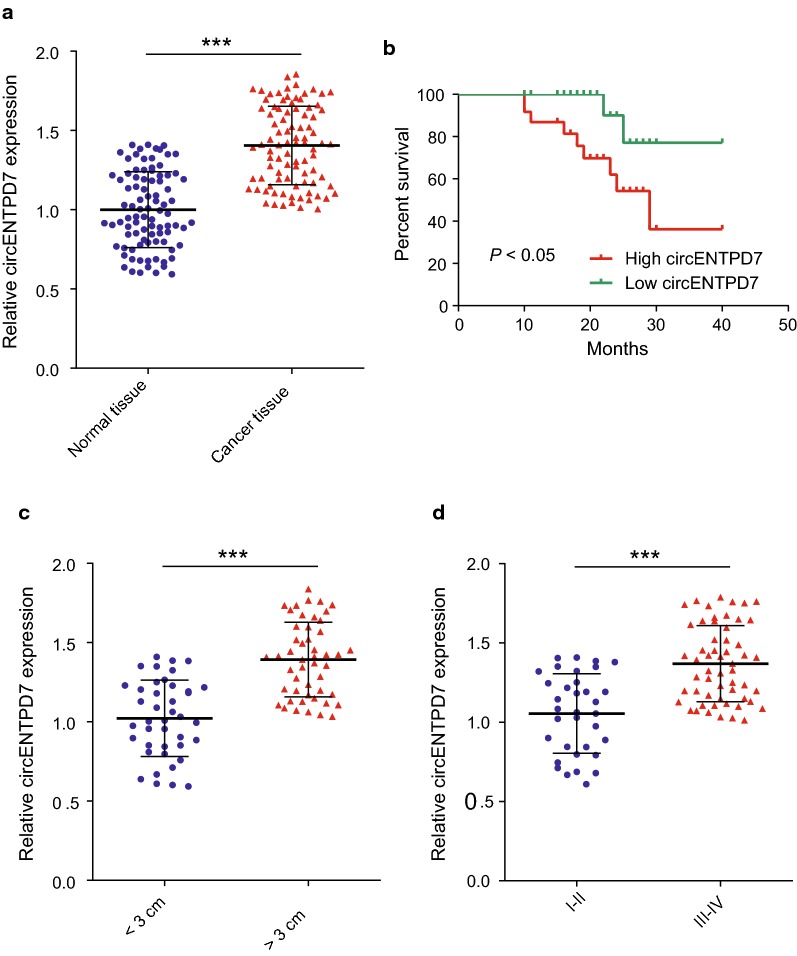
The upregulation of circENTPD7 in osteosarcoma predicts poor prognosis. **a** RT-qPCR analysis was carried out to detect the expression level of circENTPD7 in 90 glioblastoma tissues (n = 90) and paired noncancerous tissues (n = 90). **b** Kaplan–Meier univariate analysis of overall survival in glioblastoma patients with high (above median) versus low (below median) circENTPD7 levels; *P* < 0.05 [log-rank test]. **c** The circENTPD7 was examined in glioblastoma tissues < 3 cm (n = 41) and > 3 cm (n = 49). **d** The circENTPD7 was examined in glioblastoma tissues at I–II stage (n = 34) and III–IV stage (n = 56). Data are presented as mean ± SD, Student’s *t* test, ***P < 0.001

Next, we detected the circENTPD7 expression in 90 paired glioblastoma and adjacent normal tissues. The results showed that circENTPD7 was significantly upregulated in glioblastoma tissues compared to the adjacent normal tissues (Fig. 2a). The correlations between circENTPD7 expression and clinicopathological features of glioblastoma patients were analyzed. Briefly, by using median expression values, 90 patients were divided into two groups, the high and low expression groups, depending on the fold change (2^−ΔΔCT^). The results indicated that the significant high levels of circENTPD7 in patients were correlated with advanced classification and tumor size (Table 1). Additionally, the overall survival information was followed up from the patients previously and then analyzed by using the Kaplan–Meier method through GraphPad Prism software (8.0.1). It showed that patients who had high levels of circENTPD7 within their glioblastoma tissues had significant shorter overall survival (Fig. 2b). Additionally, circENTPD7 was upregulated in glioblastoma tissues that are larger than 3 cm (Fig. 2c), and also was increased in the group of glioblastoma tissues in advanced stages (Fig. 2d), implying the positive association of circENTPD7 expression with glioblastoma tumor progression and metastasis.

**Table 1:** Correlation between circENTPD7 expression and clinicopathologic characteristics of gastric cancer patients

Characteristics	circENTPD7 expression			
	Cases	High	Low	*P*
Gender				
Male	50	22	28	0.74
Female	40	19	21	
Age (years)				
< 45	31	13	18	0.26
≥ 45	59	32	27	
Family history of cancer				
Yes	13	6	7	0.89
No	77	34	43	
Tumor location				
Supratentorial	64	33	31	0.38
Infratentorial	26	16	10	
Tumor size (cm)				
< 3	41	14	27	0.02*
> 3	49	29	20	
WHO grade				
I–II	34	11	23	0.01*
III–IV	56	33	23	

### Knockdown circENTPD7 represses cell proliferation and motility of glioblastoma cells

**Fig. 3:**
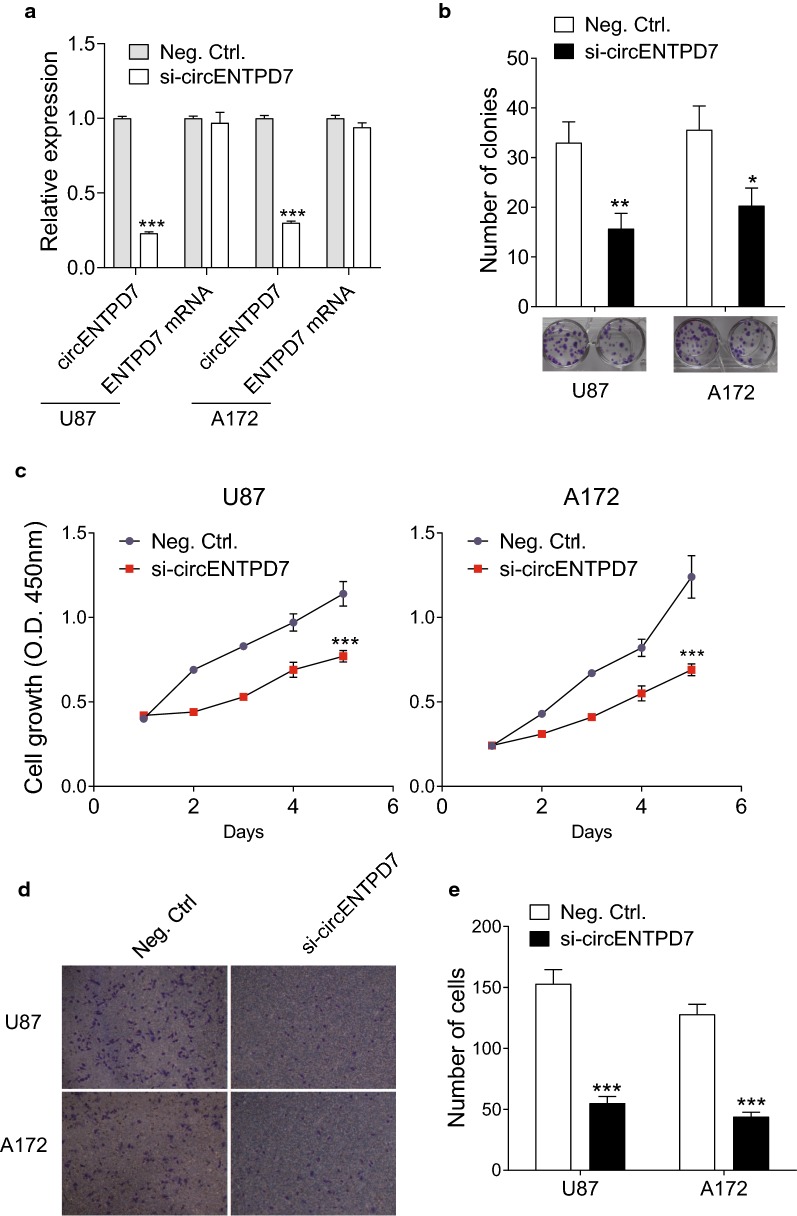
circENTPD7 knockdown represses the proliferation and metastasis of glioblastoma cells. **a** Transfection efficiency si-circENTPD7 into glioblastoma cells (U87, A172) was examined by RT-qPCR. **b** Clone formation assay demonstrated the clone number in the circENTPD7 knockdown transfection group and the control transfection. **c** CCK-8 assay showed the inhibition of circENTPD7 knockdown on the proliferation ability. **d** Transwell assay demonstrated the circENTPD7 knockdown for the invasion of glioblastoma cells comparing to the control transfection. **e** The statistical results of **d**. Data are presented as mean ± SD, Student’s *t* test, *P < 0.05, **P < 0.01, ***P < 0.001

To investigate the role of circENTPD7 in glioblastoma cells, we used siRNA to knockdown circENTPD7 in glioblastoma cells (U87, A172) (Fig. 3a). The results of clone formation assay showed a reduced number of clones in the circENTPD7 knockdown transfection group (Fig. 3b). Next, CCK-8 assay was performed to evaluate the effect of circENTPD7 knockdown on cell proliferation. Glioblastoma cells transfected with circENTPD7 siRNAs had an inhibitory effect on cell proliferation (Fig. 3c). We performed transwell migration assay to determine if circENTPD7 regulates the motility of glioblastoma cells. As shown in Fig. 3d and e, the motility of glioblastoma cells was significantly inhibited by circENTPD7 siRNAs. These results suggested that circENTPD7 knockdown represses the proliferation and metastasis of glioblastoma cells.

### circENTPD7 targets miR-101-3p as a miRNA sponge

**Fig. 4:**
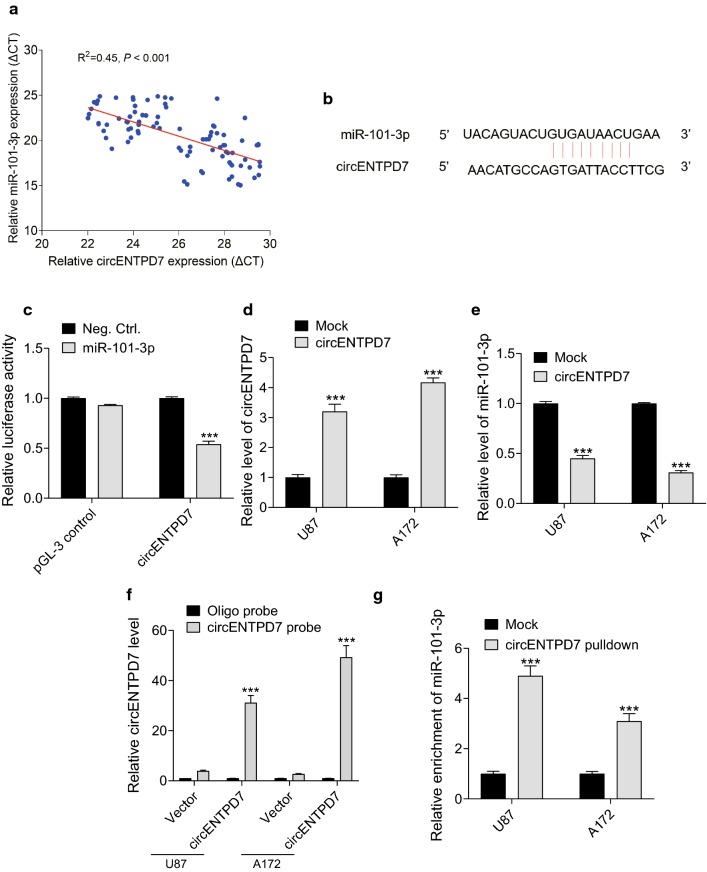
circENTPD7 targets miR-101-3p as a miRNA sponge. **a** Correlation analysis of expression of circENTPD7 and miR-101-3p in glioblastoma tissues were analyzed. **b** miR-101-3p had the complementary sites with the circENTPD7. **c** The luciferase activity on behalf of the molecular binding of circENTPD7 and miR-101-3p was tested. **d** CircENTPD7 levels in the overexpressing glioblastoma cells were tested using RT-PCR. **e** CircENTPD7 negatively regulated expression of miR-101-3p in glioblastoma cells. **f** Lysates from U87 and A172 cells with circENTPD7 vector were subjected to biotinylation-cirENTPD7 pull down assay, and expression levels of circENTPD7 were measured by RT-qPCR. **g** miR-101-3p were measured by RT-qPCR in **f**. Data are presented as mean ± SD, Student’s *t* test, ***P < 0.001

To elucidate circRNA–miRNA interaction potentials, bioinformatics databases TargetScan (https://www.targetscan.org/) and circinteractome (https://circinteractome.nia.nih.gov/) were used to predict potential binding sites of miRNAs in circENTPD7 [15, 16]. The results of this study found that miR-101-3p may be the target of circENTPD7. Then, we examined the expression (ΔCT of miR-101-3p and circENTPD7) correlations between circENTPD7 and miR-101-3p, the results showed that circENTPD7 was inversely correlated with miR-101-3p (Fig. 4a). The miR-101-3p complementary binding site to circENTPD7 was shown in Fig. 4b. The luciferase activity assay showed the molecular interaction between circENTPD7 and miR-101-3p (Fig. 4c). Next, we overexpressed circENTPD7 in U87 and A172 cells (Fig. 4d) and found that circENTPD7 could reduce the expression of miR-101-3p (Fig. 4e). To detect the interaction between circENTPD7 and miR-101-3p, the RNA–RNA pulldown assay was carried out and we found that miR-101-3p was highly enriched by circENTPD7 pulldown (Fig. 4f and 4g). Together, these results suggested that circENTPD7 serves as a miRNA sponge for miR-101-3p.

### ROS1 serves as the target of circENTPD7/miR-101-3p

**Fig. 5:**
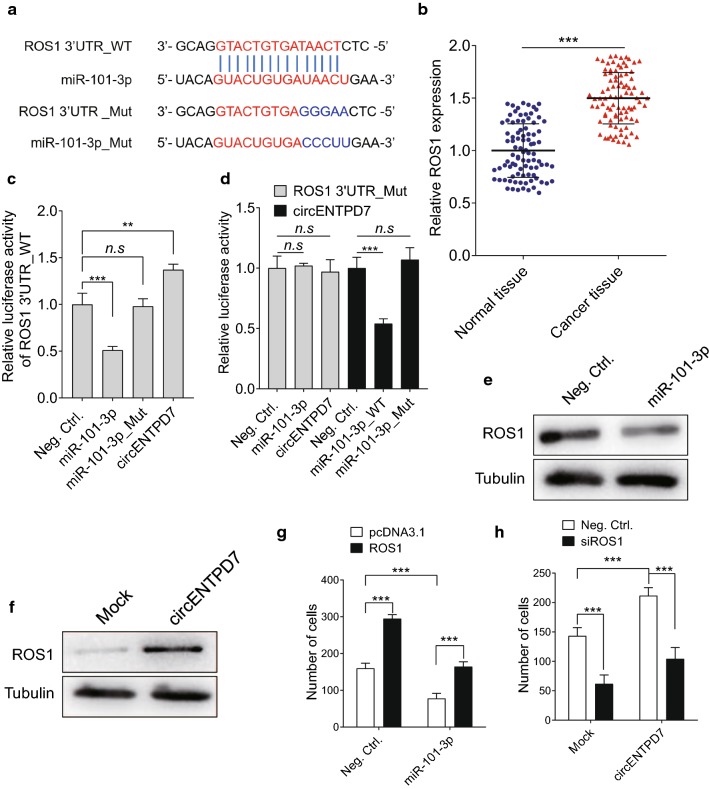
ROS1 serves as the target of miR-101-3p. **a** Diagram of 3′UTR of ROS1 containing wild type and mutations binding sites of miR-101-3p. **b** The expression of ROS1 in the glioblastoma were examined. **c** Expression of miR-101-3p mimics decreased luciferase activities in the cells transfected with plasmids containing wild type 3′UTR of ROS1, while expression of circENTPD7 increased luciferase activities in that; miR-101-3p_mut had no effect on the luciferase activity of ROS1 3′UTR. **d** miR-101-3p and circENTPD7 had no effect on the luciferase activity of ROS1 3′UTR_Mut; miR-101-3p_mut had no effect on the luciferase activity of circENTPD7. **e** Expression of miR-101-3p mimics decreased protein levels of ROS1 in glioblastoma cells. **f** Expression of circENTPD7 increased protein levels of ROS1 in glioblastoma cells. **g** Transwell assay demonstrated ROS1 was essential  miR-101-3p inhibited cell migration. **h** Transwell assay demonstrated ROS1 was essential for circENTPD7 induced cell migration. Data are presented as mean ± SD, Student’s *t* test, **P < 0.01; ***P < 0.001

Further experiments were carried out to identify the downstream target of circENTPD7 and miR-101-3p. Bioinformatics analysis with several programs including TargetScan, RNAhybrid, Findtar, and Pita, were then performed to predict the putative miR-101-3p targets. The ROS1 mRNA 3′UTR was predicted to have complementary sites with miR-101-3p (Fig. 5a). Furthermore, the levels of ROS1 in glioblastoma tissues was significantly higher than normal tissues (Fig. 5b). Then, luciferase reporter assay confirmed that miR-101-3p suppressed the expression of ROS1 and cir ENTPD7 while a mutant mimic of miR-101-3p lacking the seed sequence did not (Fig. 5c and d). Western blot analysis revealed that the expression of ROS1 was inhibited after transfecting with miR-101-3p (Fig. 5e). Further Western blot illustrated that ROS1 expression was increased in the circENTPD7 overexpressing group (Fig. 5f), indicating that circENTPD7 was at the upstream of miR-101-3p to regulate ROS1. We next transfected miR101-3p-expressing cells with ROS1 plasmid to determine whether ROS1 was required for miR101-3p inhibition of cell motility. The results showed that ROS1 significantly rescued miR101-3p-inhibited cell migration (Fig. 5g). Similarly, knockdown of ROS1 significantly decreased circENTPD7 induced cell migration (Fig. 5h). Taken together, these results suggest that ROS1 serves as the functional protein of circENTPD7/miR-101-3p.

To investigate whether ROS1 contributes to circENTPD7, we knocked-down ROS1 in U87 and A172 cells (Additional file 1: Fig. S1a). We found that circENTPD7 was decreased after interfering ROS1 (Additional file 1: Fig. S1b). Thus, ROS1-circENTPD7 feedback contributes to glioma pathogenesis. Although miR-101-3p had been reported in glioblastoma [17–19], whether miR-101-3p regulates cell growth in vivo remains unclear. We found that miR-101-3p not only inhibited cell growth in vivo (Additional file 2: Fig. S2a–c), but also decreased ROS1 expression (Additional file 2: Fig. S2d).

### Knockdown circENTPD7 inhibits tumor growth through miR-101-3p/ROS1 axis

**Fig. 6:**
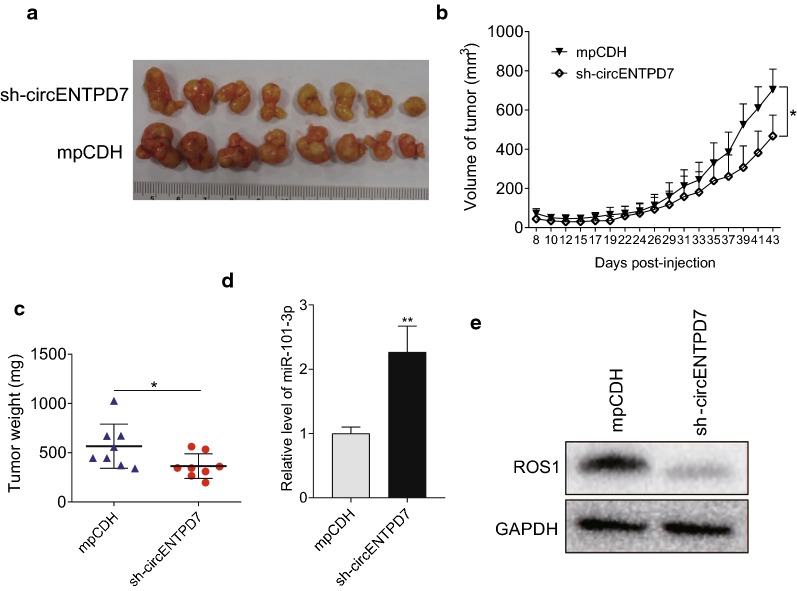
knockdown circENTPD7 inhibited tumor growth *in vivo*. **a** Representative picture showing excised tumors from circENTPD7 knockdown group (sh-circENTPD7) were much smaller than the negative control group. **b** Tumor growth curves of the excised tumors were shown. **c** Tumor weight of the excised tumors were shown. **d** Expression of miR-101-3p was much higher in sh-circENTPD7 group than in the control group measured by RT-qPCR. **e** Expression of ROS1 was much lower in sh-circENTPD7 group than in the control group measured by western blot. Representative images of western blot were shown. Data are presented as mean ± SD, Student’s *t* test, *P < 0.05, **P < 0.01

Then, we established glioblastoma cancer xenograft model using BALB/c nude mice. Tumors from the U87 cells transducted with sh-circENTPD7 grew much slower than the cells transducted with the control mpCDH group (mpCDH is a vector that we used to transient transfect or stable transduce shRNA into host cells) (Fig. 6a–c). Furthermore, we measured the RNA levels of miR-101-3p and protein levels of ROS1 in tumors. The expression levels of miR-101-3p were much higher in sh-circENTPD7 group compared with control group (Fig. 6d), while ROS1 was much lower in sh-circENTPD7 group (Fig. 6e). Taken together, these results indicated that circENTPD7 function as an oncogene and knockdown circENTPD7 could inhibit tumor growth by upregulation miR-101-3p which in turn decreasing ROS1.

## Discussion

Long noncoding RNA (lncRNAs) and circular RNAs (circRNAs) are important factors in human cancer pathogenesis [15, 20, 21]. Both lncRNAs and circRNAs lack the ability to encode proteins and circRNAs are characterized by the covalent conjunction and lacking of the 3′ and 5′ end. In glioblastoma, circRNAs have been found to exert an oncogenic or anti-oncogenic role in tumorigenesis [22, 23].

In the present study, dysregulated circRNAs were identified in glioblastoma tissue and circENTPD7 was found to be significantly up-regulated. Five overexpressed circRNAs were identified, including circENTPD7 (hsa_circ_0019421), hsa_circ_0003026, hsa_circ_0040705, hsa_circ_0040708 and hsa_circ_0040719. Another five under-expressed circRNAs were also identified, including hsa_circ_0040723, hsa_circ_0000722, hsa_circ_0040738, hsa_circ_0040733 and hsa_circ_0007361.

Functional cellular experiments indicated that circENTPD7 silencing inhibited the glioblastoma cell motility. CircRNAs can target miRNAs by acting as miRNA sponge and binding with the RNA binding protein (RBP) to exert their function. Mechanical investigation indicated that circENTPD7 targeted miR-101-3p as a miRNA sponge, which was confirmed using luciferase reporter assay and western blotting.

The ROS1 gene belongs to the subfamily of tyrosine kinase insulin receptor genes. ROS1 is now recognized as a distinct molecular target in non-small cell lung cancer [24, 25]. But the role of ROS1 in glioblastoma still elusive. We found that ROS1 was increased in glioblastoma, knockdown ROS1 inhibited cell proliferation and motility.

The role of circRNAs in human cancers has been established in previous studies [15, 20]. For example, circPTN sponges miR-145-5p/miR-330-5p to promote proliferation and stemness in glioblastoma [26]; FUS/circ_002136/miR-138-5p/SOX13 feedback loop regulates angiogenesis in glioblastoma [27]. All these studies suggest that circRNAs target the miRNA as a miRNA sponge, and bind to its target and modulate the cellular function.

Glioblastoma, also known as glioblastoma multiforme (GBM), is the most common high grade and aggressive malignant brain tumor in adults [28]. Many circRNAs had been shown to be associated with the pathological grade of gliomas [29–31]. Thus, further studies will be necessary to reveal the molecular mechanisms underlying the role of circENTPD7 in the pathological grade of gliomas.

Taken together, this study identified the role of circENTPD7 in glioblastoma cells via sponging miR-101-3p to initiate ROS1 potential. This research characterized the regulation of circENTPD7/miR-101-3p/ROS1 axis and its role in glioblastoma.

## Conclusion

We here provide evidence that miR-101-3p can inhibit glioblastoma pathogenesis by sponging circPENTPD7 and ROS1. Therefore, this study also provides new insights into the roles and regulatory mechanisms of miRNAs in glioblastoma pathogenesis. Our results suggest that therapeutic approaches targeting miR-101-3p could be useful in the treatment of glioblastoma pathogenesis.

## Supplementary information

**Additional file 1:** Figure S1 knockdown ROS1 inhibits circENTPD7. a. ROS1 was interfered in U87 and A172 cells. Representative images of western blot were shown. b. CircENTPD7 were decreased after interfering ROS1 using RT-qPCR. Data are presented as mean ± SD, Student’s *t* test, ***P < 0.001.

**Additional file 2:** Figure S2 miR-101-3p inhibits cell growth in vivo. a. Representative picture showed that miR-101-3p inhibits cell growth in vivo. b. Tumor growth curves of the excised tumors were shown. c. Tumor weight of the excised tumors were shown. d. Expression of ROS1 was much lower in miR-101-3p group than in the control group measured by western blot. Representative images of western blot were shown. Data are presented as mean ± SD, Student’s *t* test, **P < 0.01.

## Data Availability

The data sets used and/or analyzed during the current study are available from the corresponding author upon reasonable request.

## Abbreviations

circRNA: Circular RNA
DMEM: Dulbecco’s modified Eagle’s medium
FBS: Fetal bovine serum
GBM: Glioblastoma multiforme
